# Incretin Analogues for Weight Reduction in Non-Diabetic Obese: A Review of Liraglutide, Semaglutide, and Tirzepatide Beyond Glycemic Control

**DOI:** 10.5041/RMMJ.10565

**Published:** 2026-01-28

**Authors:** Krishna Nikhila Bonga, Milan Padhan

**Affiliations:** 1 Department of Pharmacology, All India Institute of Medical Sciences (AIIMS), Bhubaneswar, India; 2 Department of Clinical Pharmacology, Jawaharlal Institute of Postgraduate Medical Education and Research (JIPMER), Puducherry, India

**Keywords:** Liraglutide, obesity, semaglutide, tirzepatide, weight reduction

## Abstract

Obesity is a complex, multifactorial disease that contributes to a broad range of cardiometabolic, reproductive, and psychological disorders. Representing a major global health challenge, obesity can be addressed by lifestyle modifications such as reduced calorie intake, physical activity, adequate sleep, and stress management to help achieve sustainable weight loss and improve metabolic health in the long term. Glucose-dependent insulinotropic polypeptide (GIP) and glucagon-like peptide-1 (GLP-1) are the two naturally produced incretin hormones in the gastrointestinal tract. Incretin analogues were initially approved for type 2 diabetes mellitus but were later found to exhibit weight-reducing properties. Liraglutide, semaglutide, and tirzepatide are the three incretin analogues approved for obesity in non-diabetic patients. This narrative review presents detailed comparisons of the three approved incretin analogues for obesity, their cost-effectiveness, and trends in the clinical setting.

## INTRODUCTION

Obesity is a complex multifactorial disease characterized by abnormal or excessive fat accumulation that poses health risks associated with increased morbidity and mortality.^1^ A body mass index over 30 kg/m^2^ is defined as obesity.^2^ It affects almost all organ systems, leading to numerous comorbid conditions and diminished quality of life. It is associated with a wide range of complications, including type 2 diabetes mellitus (T2DM), hypertension, dyslipidemia, cardiovascular disease, stroke, obstructive sleep apnea, non-alcoholic fatty liver disease, osteoarthritis, gastroesophageal reflux disease, gallstones, polycystic ovary syndrome, infertility, certain cancers, and psychological conditions such as depression and anxiety.^1^,^3^,^4^

Lifestyle modifications such as reducing calorie intake, engaging in physical activity, obtaining adequate sleep, and managing stress help achieve sustainable weight loss and improve metabolic health in the long term.^5^,^6^ Pharmacological medications like orlistat, phentermine-topiramate, phentermine, naltrexone-bupropion, and some incretin analogues have been approved in obesity management.^7^ Glucose-dependent insulinotropic polypeptide (GIP) and glucagon-like peptide-1 (GLP-1) are the two naturally produced incretin hormones in the gastrointestinal tract.^8^ Incretin analogues were initially approved for T2DM and showed a weight reduction property.^1^

Intragastric balloon systems, bariatric endoscopic surgery, gastric emptying (aspiration) systems, and hydrogels may be considered for patients who do not tolerate or respond to or who prefer to avoid medications; those who are unwilling or unsuitable for bariatric surgery; or as a bridging option prior to surgery.^9^,^10^

Liraglutide, semaglutide, and tirzepatide are the three incretin analogues approved for obesity in non-diabetic patients.^8^ This narrative review presents detailed comparisons of the three approved incretin analogues for obesity and their cost effectiveness.

## OVERVIEW OF INCRETIN ANALOGUES FOR WEIGHT REDUCTION

A comparative analysis of the pharmacological similarities and differences among liraglutide, semaglutide, and tirzepatide is presented in Table 1.

**Table 1:** Comparison of Liraglutide, Semaglutide, and Tirzepatide.^11^–^23^

Parameter	Liraglutide	Semaglutide	Tirzepatide
Drug class	GLP-1 receptor agonist	GLP-1 receptor agonist	Dual GIP and GLP-1 receptor agonist
Year of approval (US-FDA)	2014	2021	2023
Route	Subcutaneous	Subcutaneous	Subcutaneous
Approved in adolescents	Approved (≥12 years age)	Approved (≥12 years age)	Not yet approved
Frequency	Daily	Weekly	Weekly
Dosing	3.0 mg	2.4 mg	Starting at 2.5 mg, titrated up to 15 mg
Mechanism of action	Activates GLP-1 receptors → enhances insulin secretion, suppresses glucagon, slows gastric emptying, promotes satiety	Activates GLP-1 receptors → enhances insulin secretion, suppresses glucagon, slows gastric emptying, promotes satiety	Activates both GIP and GLP-1 receptors → synergistic effect on insulin secretion, satiety, and weight loss
Half-life (t 1/2)	~13 hours	~7 days	~5 days
Average weight loss (%)	5%–8%	12%–15%	15%–20%
Glycemic control (HbA1c reduction)	1.0%–1.5%	1.5%–1.8%	2.0%–2.4%
Adverse effects	Primarily gastrointestinal	Primarily gastrointestinal	Primarily gastrointestinal
Contraindications	Personal or family history of MTC; patients with MEN 2; and pregnancy	Personal or family history of MTC; patients with MEN 2; and pregnancy	Personal or family history of MTC; patients with MEN 2; and pregnancy
Other approved indications	T2DM Cardiovascular risk reduction	T2DM Cardiovascular risk reduction	T2DM
Cardiovascular outcome trials	LEADER: ↓ MACE risk in T2DM	SUSTAIN and SELECT: ↓ CV events in T2DM and obesity	SURPASS-CVOT: ongoing
Obesity trials	STEP 8 and SCALE	STEP 1, STEP 3, STEP 4, and STEP 8	SURMOUNT 1 and SURMOUNT 3
Monthly cost (US dollars)	~$1,349	$998–$1,400 (depending on dose/brand)	$1,000–$1,500
US boxed warning	Thyroid C-cell tumors (based on rodent studies)	Thyroid C-cell tumors (based on rodent studies)	Thyroid C-cell tumors (based on rodent studies)

CV, cardiovascular; CVOT, cardiovascular outcomes trial; GIP, glucose-dependent insulinotropic polypeptide; GLP-1, glucagon-like peptide-1; HbA1c, hemoglobin A1c; MACE, major adverse cardiovascular events; MEN 2, multiple endocrine neoplasia type 2; MTC, medullary thyroid carcinoma; T2DM, type 2 diabetes mellitus.

### Clinical Evidence for Weight Loss in Non-diabetic Obese

Evidence from major randomized trials has demonstrated clinically meaningful weight loss with liraglutide, semaglutide, and tirzepatide in non-diabetic adults.

#### Liraglutide

In the 32-week SCALE trial, liraglutide 3 mg led to a significantly greater mean percentage weight loss compared to placebo (−5.7% versus −1.6%), along with a greater reduction in the apnea–hypopnea index (−12.2 versus −6.1 events/hour) in individuals with obesity and moderate or severe obstructive sleep apnea.^11^

#### Semaglutide

Semaglutide has demonstrated significant weight reduction in multiple clinical trials. In the 68-week STEP 1 trial, participants receiving a once-weekly 2.4 mg dose of semaglutide experienced a mean weight change of −14.9%, compared to −2.4% with placebo.^12^ Similarly, the STEP 3 trial reported a mean weight change of −16.0% with semaglutide versus −5.7% with placebo.^13^ In the STEP 4 trial, participants who continued semaglutide after a 20-week run-in period maintained further weight change (−7.9%) by week 68, whereas those switched to placebo regained weight, showing a mean change of +6.9%.^14^

#### Semaglutide versus Liraglutide

In the 68-week STEP 8 trial, a statistically significant reduction in body weight was observed, with a mean percentage change of −15.8% for once-weekly semaglutide 2.4 mg and −6.4% for once-daily liraglutide 3 mg.^15^

#### Tirzepatide

In the 72-week SURMOUNT 1 trial, tirzepatide resulted in mean weight change of −15.0%, −19.5%, and −20.9% with weekly doses of 5 mg, 10 mg, and 15 mg, respectively, compared to −3.1% with placebo.^16^ Similarly, in the 72-week SURMOUNT 3 trial, tirzepatide at its maximum tolerated dose (MTD) of 10 or 15 mg achieved a statistically significant mean weight change of −18.4%, compared to a −2.5% change with placebo.^17^

### Safety

The overall safety profile of these agents is generally favorable, with most adverse effects involving the gastrointestinal system (nausea, vomiting, diarrhea, constipation, and headache). A high incidence of gastrointestinal adverse effects has been reported, which was dose-dependent. Based on animal studies, all these drugs have a boxed warning for a potential risk of thyroid C-cell tumors, including medullary thyroid carcinoma.^24^,^25^

### Off-label and Emerging Uses

Beyond their approved indications, incretin analogues have been investigated for various metabolic and endocrine conditions. Off-label use has been proposed for several conditions, including polycystic ovary syndrome,^26^,^27^ non-alcoholic fatty liver disease,^28^ prediabetes and metabolic syndrome,^29^,^30^ obstructive sleep apnea,^11^,^31^ weight regain after bariatric surgery,^32^ and cardiovascular diseases,^33^ including heart failure with preserved ejection fraction.^34^,^35^

### Oral Semaglutide for Obesity

Recent efforts have focused on developing an oral semaglutide to improve accessibility and patient compliance. Oral peptides have poor bioavailability due to their enzymatic degradation in the intestine. Sodium N-(8-[2-hydroxybenzoyl] amino) caprylate (SNAC), an intestinal permeation enhancer, adheres to the gastric mucosa, enabling the effective oral delivery of peptides like semaglutide. The oral formulation of semaglutide utilizes SNAC technology to enhance its absorption in the stomach and protect it from degradation by gastric enzymes.^36^,^37^ Oral semaglutide has been approved only for T2DM and is not yet approved for obesity.

### Spending and Real-world Utilization

The growing use of these medications has led to significant changes in prescription trends and healthcare spending. Between 2018 and 2023, total US spending on GLP-1 receptor agonists rose by over 500%, climbing from $13.7 billion to $71.7 billion. During this period, spending on liraglutide increased from $0.56 billion to $0.89 billion. Meanwhile, from 2021 to 2023, spending on semaglutide surged from $0.58 billion to $6.99 billion.^38^

### Practical Considerations in Clinical Use

Clinicians should consider factors such as cost, tolerability, and patient adherence when prescribing these agents. Cost and insurance coverage should be evaluated, as affordability often determines long-term adherence. The therapeutic benefit of GLP-1 receptor agonists in promoting satiety is closely linked to their ability to delay gastric emptying, resulting in gastric distension. While this contributes to weight loss, it frequently causes nausea, vomiting, and other gastrointestinal symptoms. Constipation, diarrhea, and, in some cases, gallstone formation have also been described due to altered intestinal and biliary motility. These adverse effects are clinically relevant, as they often affect treatment compliance and may necessitate dose adjustment or discontinuation.^39^–^42^ Patients must be educated about expected gastrointestinal adverse effects, which often subside with time. Studies have shown that when combined with lifestyle modifications, such as calorie restriction and increased physical activity, these agents can sustain long-term weight loss.^43^,^44^ The disadvantages of losing muscle mass during weight loss are being recognized, and work is underway on ways to preserve muscle mass while losing fat. Animal studies have suggested that antibody blockade of activin type II receptors may preserve lean mass and increase fat loss during treatment with GLP-1 receptor agonists.^45^

### Is Weight Loss Maintained after Stopping?

The durability of weight loss following treatment discontinuation remains an important clinical question. These incretin analogues reduce appetite and food intake. However, upon discontinuation, appetite typically increases, often leading to weight regain. In the STEP 1 extension trial, patients who stopped semaglutide regained approximately two-thirds of the weight they had lost within a year. Since all these drugs act through a similar mechanism, the risk of weight regain remains.^46^ Therefore, continued use of the medication as prescribed by a physician is essential for sustained weight management.

### Ethical and Societal Considerations

The expanding use of GLP-1 receptor agonists beyond their approved indications raises important ethical and societal concerns. While these agents offer substantial clinical benefits for individuals with obesity or T2DM, their use among populations that are not medically ill, such as those seeking cosmetic weight reduction, presents moral and distributive challenges. With monthly list prices often in the range of US$1,000–$1,500, these drugs remain unaffordable for many uninsured or low-income patients, thereby widening existing health disparities. In addition, the widespread off-label or lifestyle-driven use of GLP-1 receptor agonists can contribute to drug shortages, limiting access for patients with established medical indications such as T2DM. The rapid commercialization and pervasive social-media promotion of GLP-1 receptor agonists raise further moral questions surrounding the medicalization of normal body image, the potential reinforcement of obesity-related stigma, and the influence of pharmaceutical marketing on prescribing behaviors.^38^,^47^,^48^

## FUTURE DIRECTIONS AND RESEARCH GAPS

Research is ongoing to explore the use of these drugs in additional indications such as cardiovascular disease, chronic kidney disease, and non-alcoholic steatohepatitis, potentially leading to expanded therapeutic applications in the future. While oral semaglutide is currently approved for T2DM, clinical trials are underway to evaluate its effectiveness in treating obesity. In the TRIUMPH-1 trial, retatrutide, a triple agonist targeting GLP-1, GIP, and glucagon receptors, demonstrated ~24% body weight loss over 48 weeks, suggesting it may surpass tirzepatide in weight reduction.^49^ Additionally, the REDEFINE trial showed that the combination of semaglutide with cagrilintide (an amylin analogue) resulted in enhanced weight loss compared to semaglutide alone.^50^

## CONCLUSION

To conclude, the evolution of incretin analogues for obesity has led to increasingly potent options for weight loss, progressing from liraglutide through semaglutide to tirzepatide. While all offer valuable therapeutic benefits, the greater efficacy of the newer, weekly-administered agents, semaglutide and tirzepatide, coupled with their potentially lower overall cost, marks a significant advancement in obesity pharmacotherapy.

## Abbreviations

GIP: glucose-dependent insulinotropic polypeptide
GLP-1: glucagon-like peptide-1
SNAC: sodium N-(8-[2-hydroxybenzoyl] amino) caprylate
T2DM: type 2 diabetes mellitus.
